# Facing Life in Old Age: Exploring Resilience in Older Adults with Bipolar Disorder

**DOI:** 10.3390/jcm13133942

**Published:** 2024-07-05

**Authors:** Laura Montejo, Mònica Retuerto, Brisa Solé, Sara Martín, Andrea Ruiz, Derek Clougher, Marta Bort, Jose Sánchez-Moreno, Anabel Martínez-Arán, Eduard Vieta, Carla Torrent

**Affiliations:** 1 Bipolar and Depressive Disorders Unit, Hospital Clinic de Barcelona. c. Villarroel, 170, 08036 Barcelona, Spain; lmontejo@recerca.clinic.cat (L.M.);; 2 Institut d’Investigacions Biomèdiques August Pi i Sunyer (IDIBAPS), c. Villarroel, 170, 08036 Barcelona, Spain; 3 Institute of Neurosciences (UBNeuro). c. Casanova, 143, 08036 Barcelona, Spain; 4 Centro de Investigación Biomédica en Red de Salud Mental (CIBERSAM), Instituto de Salud Carlos III, 28029 Madrid, Spain; 5 Departament de Psicologia Clínica i Psicobiologia, Facultat de Medicina i Ciències de la Salut, Universitat de Barcelona (UB), c. Casanova, 143, 08036 Barcelona, Spain; 6 Departament de Medicina, Facultat de Medicina i Ciències de la Salut, Universitat de Barcelona (UB), c. Casanova, 143, 08036 Barcelona, Spain

**Keywords:** bipolar disorder, OABD, elderly, age of onset, resilience, functioning, cognitive reserve, psychiatry

## Abstract

**Background:** Older adults with bipolar disorder (OABD) are individuals aged 50 years and older with bipolar disorder (BD). People with BD may have fewer coping strategies or resilience. A long duration of the disease, as seen in this population, could affect the development of resilience strategies, but this remains under-researched. Therefore, this study aims to assess resilience levels within the OABD population and explore associated factors, hypothesizing that resilience could improve psychosocial functioning, wellbeing and quality of life of these patients. **Methods**: This study sampled 33 OABD patients from the cohort at the Bipolar and Depressive Disorders Unit of the Hospital Clinic of Barcelona. It was an observational, descriptive and cross-sectional study. Demographic and clinical variables as well as psychosocial functioning, resilience and cognitive reserve were analyzed. Resilience was measured using the CD-RISC-10. Non-parametric tests were used for statistical analysis. **Results:** The average CD-RISC-10 score was 25.67 points (SD 7.87). Resilience negatively correlated with the total number of episodes (*p* = 0.034), depressive episodes (*p* = 0.001), and the FAST (*p* < 0.001). Participants with normal resilience had a lower psychosocial functioning (*p* = 0.046), a higher cognitive reserve (*p* = 0.026), and earlier onset (*p* = 0.037) compared to those with low resilience. **Conclusions:** OABD individuals may have lower resilience levels which correlate with more psychiatric episodes, especially depressive episodes and worse psychosocial functioning and cognitive reserve. Better understanding and characterization of resilience could help in early identification of patients requiring additional support to foster resilience and enhance OABD management.

## 1. Introduction

Bipolar disorder (BD) is a chronic and recurrent affective disorder characterized by mood and energy fluctuations, which include manic, hypomanic and depressive episodes, along with significant subsyndromal symptoms. BD is one of the main causes of disability and it is associated with premature death due to high rates of suicide and physical comorbidities [1,2].

It has also been described that multiple dimensions of aging seem to be altered in BD, leading to a premature aging process [3]. Older Adults with Bipolar Disorder (OABD) refers to patients older than 50 years with BD [4,5]. Approximately 25% of all patients with BD are older than 60 and this number is expected to increase to 50% in 2030 [6]. With the rapid aging of the world’s population and the increased life expectancy of people with chronic health conditions such as BD, there is an urgent need to better characterize OABD [7,8].

In general, the clinical course of BD is understudied in OABD. Recently, findings from the Global Aging & Geriatric Experiments in Bipolar Disorder (GAGE-BD) project suggest some changes in the clinical pattern during the aging process. For instance, while some clinical features appear to be less severe (such as manic episodes and psychotic symptoms) [9,10], other factors emerge as more prominent; such as suicide attempts, depressive symptoms, mixed episodes, somatic comorbidities, premature death, impairment in psychosocial functioning and cognitive dysfunction or dementia [10,11,12,13,14,15]. In addition, some reports have detected differences according to the age of onset (early vs. late), in which late onset showed poorer cognitive outcomes and higher cerebrovascular risk [16]. Thus, OABD constitutes a more complex population due to the long-term effects of the disease coupled with the impact of aging. Older adults may face life stressors that are common to all age groups, as well as stressors that are more typical in later life; for example, a gradual decline in functional abilities and capacities, forced retirement, caregiving, financial stress, and loss of independence [17,18]. In OABD, in addition to coping with those stressors, the impact of the disease becomes an additional factor to manage such as chronic illness, cognitive decline, somatic comorbidities, loneliness, etc.

A paradigm shift towards mental health promotion is increasingly seen as an approach to improve overall wellbeing, helping people live better with their illness [19]. A core aspect of wellbeing is resilience, which is considered one of the most important aspects of both prevention and intervention in mental illness [20,21]. Resilience refers to a person’s ability to adapt to, cope with and recuperate from a negative experience [22], which can be in the form of relationship issues, health problems, work and financial concerns, among other challenges [23]. Furthermore, resilience is seen as a dynamic multidimensional construct that not only involves personal characteristics, abilities, or skills; but also family support and other external factors [24]. Because it also includes contextual resources, which may be learned and acquired, resilience is considered a process rather than a trait [25]. Some “resilience factors” or coping mechanisms include an optimistic but realistic outlook, sturdy role models, an inner moral compass, religious or spiritual practices, acceptance of what cannot be changed, physical fitness, mental sharpness, emotional strength, actively solving problems while looking for meaning and opportunity, and humor [26].

In the context of mental illness, resilience appears to moderate the risk of depression [27], negative affect and perceived stress [28], and is also associated with a reduced suicidal ideation [29]. Among BD patients, higher levels of resilience have been associated with lower severity of clinical symptoms such as depression, psychotic symptoms or suicide attempts, but also with better social and psychosocial outcomes [21]. Thus, resilience is considered a protective factor that promotes a positive outcome among people facing adverse circumstances. It is believed that resilience may be a key factor in improving health outcomes for people with BD, in areas such as psychosocial functioning, wellbeing and quality of life [25,30].

A challenge to understand the role of resilience in OABD is that it could be impacted by the course of the disease, the illness stage, the severity and other clinically specific features. Furthermore, it is possible that the effects of the illness hinder the acquisition of coping mechanisms for life stressors, as less resilience is observed in those with BD compared to healthy controls (HC) [31]. On one hand, the long course of the disease frequently experienced by OABD could impact the development of resilience strategies, affecting their functioning, quality of life and wellbeing. On the other hand, sometimes OABD are described within the concept of a “survivor cohort”, which refers to the suggestion that OABD form a group of less severely affected patients, because those with the highest burden experience premature death. This may be related to the proposal that these older adults have acclimated to their diagnosis and symptoms, and have devised effective coping strategies [32]. Additionally, it has been noted that patients with BD who reach advanced age may even have a less severe phenotype of the disease, displaying better conditions to develop better levels of resilience and coping strategies. Thus, it appears of most noticeable importance to better characterize resilience in OABD. A better understanding of resilience could be useful for an early identification of profiles of patients who may require more assistance to foster resilience and improve management of BD [21]. The aim of this study was to measure and characterize resilience in OABD, as well as to observe which factors are associated with it.

## 2. Materials and Methods

### 2.1. Design and Participants

This is an observational, descriptive and cross-sectional study. The sample for the present study was selected out of the preexisting OABD cohort from the Bipolar and Depressive Disorders Unit of the Hospital Clinic of Barcelona. The inclusion criteria were: (1) a diagnosis of BD according to the Diagnostic and Statistical Manual of Mental Disorders fifth edition (DSM-V); (2) being ≥50 years of age; (3) able to speak fluent Spanish; (4) being clinically euthymic or in partial remission during the assessment, defined as a score of ≤14 in the Hamilton Depression Rating Scale (HDRS) and a score of ≤10 in the Young Mania Rating Scale (YMRS); and (4) giving signed inform consent. The exclusion criteria were: (1) presence of any other comorbid psychiatric condition except for sleep and/or anxiety disorders; (2) presence of a central nervous system (CNS) condition such as neurological disease; (3) an Intelligence Quotient (IQ) lower than 85; and (4) having received electroconvulsive therapy in the prior six months. All patients were informed about the purpose of the study. The study was conducted in accordance with the ethical principles of the Declaration of Helsinki and Good Clinical Practice, and was approved by the Hospital Clinic Ethics and Research Board (Approval Code: HCB/2020/0116, Date: 3 March 2020).

### 2.2. Assessments

#### 2.2.1. Demographic and Clinical Assessment

The assessed demographic and clinical characteristics were current age, sex, number of years of education, work situation, type of diagnosis, total number of episodes and the number of each type of episode, type of onset (early vs. late), type of first episode, number of psychiatric admissions, years of illness duration, history of suicidal ideation and suicide attempts, family history of psychiatric disorders, and pharmacological treatment. Regarding type of onset, early onset (EOBD) was defined as patients experiencing a first episode before the age of 50 years; late onset (LOBD) was defined as those whose first symptoms and episodes occurred after the age of 50 [6].

Diagnoses were determined with the Structured Clinical Interview for DSM (SCID-I and II) [33,34] according to DSM-V criteria. Patients were assessed with two clinical scales: the Young Mania Rating Scale (YMRS) [35] for the evaluation of manic symptoms and the Hamilton Depression Rating Scales (HDRS) [36] for the depression symptoms. On each scale, the items were summed to obtain a total score. Higher scores indicate greater severity of the assessed symptomatology.

#### 2.2.2. Psychosocial Functioning

This was evaluated through the Functioning Assessment Short Test (FAST) [37]. It is a brief instrument designed to assess the main functioning problems experienced by psychiatric patients, especially bipolar patients. It includes six functional domains (autonomy, occupational functioning, cognitive functioning, financial issues, interpersonal relationships and leisure time). Higher scores indicate worse functioning. It has a highly significant negative correlation with GAF (r = −0.903; *p* < 0.001). A cutoff of 11 points obtained the best balance between sensitivity (72%) and specificity (87%). In addition, the FAST has different severity thresholds: no impairment (from 0 to 11 points); mild impairment (from 12 to 20); moderate impairment (from 21 to 40); and, finally, scores above 40 represent severe functional impairment [38].

#### 2.2.3. Cognitive Reserve (CR)

CR was evaluated through the Cognitive Reserve Assessment Scale in Health (CRASH) [39]. It is an instrument developed to measure cognitive reserve specifically in people with severe mental illness. It was validated including non-affective psychoses and affective disorders compared to healthy controls. The CRASH global score had a large positive correlation with the Cognitive Reserve Questionnaire total score (r = 0.838, *p* < 0.001). With a cutoff value of 47.58, it has the highest sensitivity (79%) and specificity (80.30%). It provides a global score and a score for each of the domains that form it (education, occupation, and intellectual and leisure activities). The scale’s maximum total score is 90, and it can be calculated using a formula, created with the intention that all domains have the same weighting in the final score. The score for each domain is obtained by adding the scores of the items it contains. For all scores, the higher the result, the better the level of CR.

#### 2.2.4. Assessment of Resilience

Resilience was measured through the Connor–Davidson Resilience Scale-10 items (CD-RISC-10) [40], which is a shorter version of the original CD-RISC [41]. It has been validated in the Spanish language [42] and for its use with non-institutionalized older adults [43]. The CD-RISC-10 consists of 10 items that form a summative Linkert type scale, in which each item can be scored from 0 (not true at all) to 4 (true nearly all the time). The total range of the scale goes from 0 to 40, without an established cutoff point; the higher the score, the higher the levels of resilience [43]. The Spanish version for older adults showed good psychometric properties, with a Cronbach’s coefficient of 0.81.

### 2.3. Statistical Analysis

The quantitative variables were described using their mean and standard deviation, and the qualitative variables were described through their frequency and percentage. Normality was assessed using the Shapiro–Wilk test. To evaluate the correlation between the CD-RISC-10 and the quantitative measures, Spearman’s correlation was used, and for the qualitative measures, the Mann–Whitney U test was used. For calculating levels of resilience, we selected the criteria set by Campbell-Sills and Stein [40], in which high values of resilience were defined as more than one standard deviation above the mean, according to normative data; and low resilience was defined as one and more standard deviation below the mean. The values between these two points were considered to be in the normal range. The description of the different groups of resilience was conducted through the Mann–Whitney U test in the case of the quantitative measures and the Chi-squared test in the case of the qualitative measures. For all analyses, a two-sided alpha of 0.05 was considered statistically significant. SPSS 25 was used for all statistical analyses.

## 3. Results

### 3.1. Sample Characteristics

A total of 33 participants were included in the study. The average age was 65.67 years, with an average of years of education of 15.21 years; and more than half of the sample was represented by women (57.6%). The average number of years of illness duration was 31.81. The total FAST and CRASH had a mean of 22.8 and 44.23, respectively. 

Regarding their diagnosis, BD type I was the most frequent. Relating to the type of onset, EOBD was more prevalent with a 84.8% of the total sample. Depression was the type of first episode that was most frequent (84.4%). When it comes to work status, most of the patients were retired or were on permanent leave. A total of 62.5% of the participants had a family history of psychiatric disorders. More than half of the sample had suicidal ideation. All sample characteristics are displayed in Table 1.

### 3.2. Evaluation of Resilience

In the evaluated group of 33 participants, the total CD-RISC-10 had a mean of 25.67 (SD = 7.87), with a minimum of 11 and a maximum of 39 points. It follows normality through the Shapiro–Wilk test (0.964), with a *p*-value of 0.340.

#### 3.2.1. Correlations of Resilience with Clinical and Psychosocial Variables

Using Spearman’s correlation to assess the relation between the CD-RISC-10 and the quantitative measures, a negative correlation was observed between resilience and the total number of episodes (r = −0.389, *p*-value = 0.034), as well as between resilience and the number of depressions (r = −0.622, *p*-value = 0.001). There was also a negative correlation between the CD-RISC-10 and the FAST scale (r = −0.61, *p*-value < 0.001). No more significant correlations were found with other variables (Table 2).

#### 3.2.2. Group Analysis of Resilience

**Figure 1:**
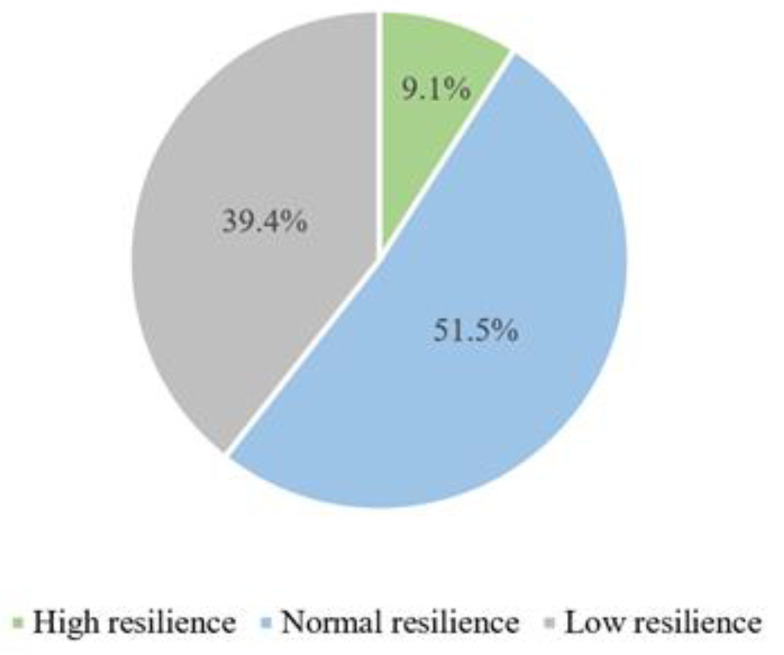
Groups of high, normal and low resilience.

According to the group classification of resilience by Campbell-Sills and Stein, out of 33 participants, 3 (9.1%) were considered to have high resilience, 17 (51.5%) had normal resilience, and 13 (39.4%) had low resilience, as shown in Figure 1.

We performed mean differences between the most represented group in terms of sample size; that is, lower and normal resilience. When comparing the group with low resilience and that with normal resilience, a significant difference in psychosocial functioning was observed. Thus, OABD patients with low resilience had a higher FAST and therefore worse functioning when compared to those with normal resilience (Mann–Whitney U test = 58; *p*-value = 0.044). A significant difference was also observed regarding CR, in which the group with low resilience had a lower CRASH score and therefore worse cognitive reserve when compared to those with normal resilience (Mann–Whitney U test = 33.5, *p*-value = 0.026). Finally, a significant difference was observed when it comes to the type of onset, with the group with normal resilience being exclusively made up by participants with EOBD and the one with low resilience being made up by participants with both EOBD and LOBD (Chi-squared test = 4.359, *p*-value = 0.037). No significant differences between the two groups were observed concerning other measures (Table 3).

## 4. Discussion

To the best of our knowledge, this is the first study to evaluate resilience in a sample of OABD. We found that resilience was correlated with some clinical factors such as the total number of episodes, particularly depressive episodes, in which more episodes indicate lower resilience. Psychosocial functioning was also significantly associated with resilience, showing that OABD with better functioning exhibited higher levels of resilience. Furthermore, the sample could be classified into three groups based on resilience levels. The group with low resilience presented worse psychosocial functioning and CR compared to the group with normal resilience. Notably, the group with normal resilience consisted only of patients with EOBD.

As previously described, resilience is a multidimensional subject that relates to an individual’s ability to adapt positively in response to significant adversity [26]. Compared to healthy controls, resilience levels in patients with BD have been described to be lower, even during euthymic periods [23]. In recent years, the CD-RISC-10 has been used in psychiatry, specifically for evaluating patients with BD; but it had never been used before to assess resilience in OABD. According to normative data, our results show that almost half of our sample exhibited low resilience, while half of the patients displayed normal resilience levels, with very few patients demonstrating high resilience.

The results of this study show a negative correlation between resilience and the total number of episodes; suggesting that with every new relapse, the resilience capacity worsens, particularly with depressive episodes. This stands in line with previous analysis, which suggests that depressive episodes have a higher negative impact on patients’ ability to manage their disease when compared to manic and hypomanic episodes. In that sense, resilience is a mediator of depressive symptoms, protecting against their onset, severity, and chronicity [44]. Patients with a long history of BD who have experienced more depressive episodes have not been able to develop coping strategies across the life span, increasing the risk of subsequent depressive episodes. Nevertheless, it is also possible that those patients with lower number of depressive symptoms have a neurobiological predisposition for stronger resilience mechanisms [45]. Another important issue is the challenge of defining resilience. Resilience has often been cited as the absence of mental illness or the maintenance of mental health in the face of adversity. However, it is crucial to broaden the perspective on this concept and consider that having a mental disorder or experiencing depressive episodes does not necessarily mean one is less resilient [46]. In fact, facing and managing such challenges often requires great strength and perseverance, demonstrating a profound level of resilience.

It has also been hypothesized that there may be an association between resilience and functioning [25], in which patients with higher resilience show better psychosocial functioning [21]. In our study, we have found a significant correlation between resilience and functioning, indicating that OABD with lower resilience also have poorer psychosocial functioning. This is further supported by the results from our resilience group analysis: participants with low resilience had lower psychosocial functioning when compared to those with normal resilience. This may be explained by the fact that resilience-determining skills such as the acceptance and understanding stressful events—such as mood episodes in OABD—and having the capacity to develop coping strategies, and enjoying a sense of belonging are crucial and necessary to function both on a personal level as well as in society. On the other hand, since functioning is also related to the number of depressive episodes, which in turn are associated with the development of resilience, it could be a multifactorial and multidirectional relationship among these constructs.

Another mental health concept that has been recently discussed is that of CR [47]. The CR hypothesis states that patients with higher IQ, education levels or occupation attainment are less likely to develop dementia [48]. CR can be defined as the ability of the brain to make flexible and efficient use of cognitive networks in order to minimize the clinical manifestations of the pathology [49], and this capacity appears to be reduced in BD patients [50]. Recent literature has proposed a possible association between resilience and CR, by complex unknown mechanisms through which resilience brain networks appear to subtend interindividual differences in terms of CR advantages [21,51]. Both concepts, CR and resilience, have been used to partially explain variable outcomes with respect to aging and disease; considering resilience the emotional aspect of CR [19]. They represent one’s capacity to use their cognitive, affective and social skills to sustain psychological stability following exposure to stressful or traumatic events. However, we have not found a significant overall correlation between these two variables; this lack of correlation may be explained by the limitations of this study, as having a small sample may cause the appearance of type II errors. Nonetheless, after analyzing the differences between patients with normal and low resilience, CR results appeared to be significantly different, with patients with low resilience having worse CR. CR can contribute to resilience by enabling individuals to better manage their symptoms and maintain cognitive functioning, thereby improving their overall quality of life and ability to cope with the challenges posed by their condition.

Additionally, as previously described, there are two major groups of OABD regarding the onset of the disease: EOBD and LOBD. There is an ongoing debate about whether these two groups vary in characteristics and if their treatments should differ [52]. So far, studies show that LOBD might represent a similar clinical phenotype as EOBD with respect to BD symptomatology, functionality and comorbid physical conditions [53]; but the question stands open of whether these two groups have different levels of resilience. This possibility was analyzed in the present study, and the results show that there is no significant overall correlation between the type of onset and resilience; but there was again a significant difference when comparing the groups, with the group with normal resilience being exclusively composed by participants with EOBD, while the group with low resilience included participants with both EOBD and LOBD, but more cases of EOBD. These findings are interesting as they can be interpreted in different ways. On the one hand, as the group with normal resilience was only composed of patients with EOBD, this subgroup of patients may have higher resilience because they have had more time to adapt and cope with their disease, as the healthy cohort hypothesis has stated. On the other hand, the higher proportion of patients with EOBD in the low-resilience group could mean that the disease itself has a negative long-term impact on resilience and coping abilities. However, it would also have been expected that at least a certain proportion of LOBD patients would achieve normal levels of resilience; as, in the absence of disease symptoms, they may have developed more resilient traits. In fact, in line with this notion, when examining the high-resilience group, we observe that two out of the three patients in this category had a late onset. Further research is needed to clarify the differences and causes regarding the association between resilience and the type of BD onset.

This study has several limitations that must be considered. First, the small sample size may limit the interpretation of the results [54]. Future studies including a larger sample size would enable more generalized and robust conclusions. Second, the average age of the patients who participated was relatively young, despite fulfilling the older age criteria. This generates doubts about the generalizability of the results to patients in the eighth and ninth decade of life and beyond, which need to be studied considering the current aging of the global population. There was also a suboptimal number of participants in the group of LOBD, which could have impacted the results regarding the type of onset. Finally, the sample size in the group with high resilience was insufficient to allow for statistically meaningful comparison with the other groups. Another limitation lies in the definition of resilience; there is not a consensus on its definition and factors included, acquiring many nuances and depending on the scale used to evaluate it. Additionally, the scale used to measure resilience is self-administered, which implies limitations associated with self-reported information which, in the context of mental illnesses, may be mediated by clinical status and cognitive difficulties. Finally, treatment was naturalistic and therefore medication might have been a confounder in some analysis [55].

To conclude, this study has evaluated resilience in OABD, and the results have shown that these patients may have lower resilience than healthy subjects in the same age group. Furthermore, an association has been observed between resilience and the total number of psychiatric episodes, and specifically the number of episodes of depression. Furthermore, the results show that patients with lower resilience have worse psychosocial functioning, lower CR and higher rates of EOBD. One of the key questions arising from this study is which patients might benefit most from therapy specifically aimed at strengthening and enhancing resilience and its coping mechanisms. Therefore, it seems it may be the group of OABD with more depressive episodes who may benefit to a greater extent from resilience-enhancing treatments, also suggesting that may subsequently improve their overall functioning and quality of life. Further studies are needed to explore resilience and clinical factors such as the severity, long-term impact or course of the disease.

## Figures and Tables

**Table 1:** Demographic and clinical characteristics of the whole sample.

	Mean (SD)	Range [Min–Max]
Age	65.67 (6.55)	[54–79]
Years of education	15.21 (2.88)	[8–19]
Number of psychiatric admissions	1.61 (2.82)	[0–15]
Total number of episodes	16.40 (16.24)	[1–81]
Number of manic episodes	1.33 (2.25)	[0–11]
Number of hypomanic episodes	6.86 (8.97)	[0–41]
Number of depressive episodes	9.63 (9.73)	[2–40]
Number of suicide attempts	1.06 (1.47)	[0–5]
Duration of illness (years)	31.81 (14.69)	[2–53]
Total YMRS	1.17 (1.94)	[0–7]
Total HDRS	5.17 (3.07)	[0–11]
Total FAST	22.8 (12.12)	[2–42]
Total CRASH	44.23 (10.61)	[19.08–72.92]
	**Frequency (%)**	
Sex (female)	19 (57.6)	
Diagnosis		
BD-I	19 (57.6)	
BD-II	11 (33.3)	
Unspecified BD	3 (9.1)	
Type of onset (early onset)	28 (84.8)	
Type of first episode		
Depression	27 (84.4)	
Hypomania	2 (6.3)	
Mania	3 (9.4)	
Employment status		
Temporary sick leave	4 (12.9)	
Permanent sick leave	12 (38.7)	
Retired	12 (38.7)	
Active	3 (9.7)	
Family history of psychiatric disorders	20 (62.5)	
Suicidal ideation	18 (58.1)	
Suicide attempts	9 (29.0)	
Pharmacological treatment		
Mood stabilizers	30 (93.8)	
Antipsychotics	21 (65.6)	
Antidepressants	14 (43.8)	
Benzodiazepines	10 (32.3)	

BD, Bipolar Disorder; CRASH, Cognitive Reserve Assessment Scale in Health; FAST, Functioning Assessment Short Test; HDRS, Hamilton Depression Rating Scale; YMRS, Young Mania Rating Scale.

**Table 2:** Correlations between the CD-RISC-10 and clinical variables.

	CD-RISC 10	
	Spearman’s Correlation	*p*-Value
Age	−0.14	0.486
Years of education	0.18	0.331
Number of psychiatric admissions	0.07	0.721
Total number of episodes	−0.39	0.034 *
Number of manic episodes	0.07	0.721
Number of hypomanic episodes	−0.33	0.148
Number of depressive episodes	−0.62	0.001 *
Number of suicide attempts	−0.16	0.532
Duration of illness (years)	−0.08	0.706
Total YMRS	−0.14	0.477
Total HDRS	−0.34	0.070
Total FAST	−0.61	<0.001 *
Total CRASH	0.28	0.164
	**Mann-Whitney U test**	***p*-value**
Sex (female)	113.50	0.477
Diagnosis	90.50	0.546
Type of onset	66.50	0.860
Family history of psychiatric disorders	108.00	0.640
Suicidal ideation	94.50	0.367
Suicide attempts	86.50	0.586
Pharmacological treatment		
Mood stabilizers	26.00	0.755
Antipsychotics	110.00	0.827
Antidepressants	111.00	0.568
Benzodiazepines	88.50	0.485

CD-RISC-10, 10 Item Connor-Davidson Resilience Scale; CRASH, Cognitive Reserve Assessment Scale in Health; FAST, Functioning Assessment Short Test; HDRS, Hamilton Depression Rating Scale; YMRS, Young Mania Rating Scale. * Indicated statistically significant values.

**Table 3:** Demographic, clinical and functional differences between participants with low and normal resilience.

		**Low Resilience** **M (SD)**	**Normal Resilience** **M (SD)**	**Mann–Whitney U Test**	***p*-Value**
Age		67.62 (6.95)	63.82 (6.45)	73.5	0.121
Years of education		14.77 (3.09)	15.71 (6.45)	89.0	0.362
Number of psychiatric hospital admissions		1.17 (1.40)	2.06 (3.70)	87.0	0.663
Total number of episodes		20.42 (21.76)	15.53 (11.40)	84.0	0.769
Number of manic episodes		1.00 (1.49)	1.64 (2.82)	58.5	0.462
Number of hypomanic episodes		9.86 (14.25)	6.36 (4.95)	38.0	0.964
Number of depressive episodes		14.36 (12.71)	7.38 (5.49)	48.0	0.169
Number of suicide attempts		0.83 (0.98)	1.30 (1.77)	27.5	0.771
Duration of illness (years)		29.55 (15.99)	27.62 (10.48)	49.0	0.192
Total YMRS		1.33 (2.19)	1.29 (1.94)	81.0	0.866
Total HDRS		5.92 (3.23)	4.80 (2.93)	69.5	0.315
Total FAST		27.54 (8.49)	19 (13.47)	58	0.044 *
Total CRASH		39.63 (10.45)	49.62 (9.66)	33.5	0.026 *
		**Low Resilience** **N (%)**	**Normal Resilience** **N (%)**	**Chi-Squared Test**	***p*-Value**
Sex (female)		8 (61.5)	10 (58.8)	0.02	0.880
Diagnosis	BD I	6 (46.2)	12 (70.6)	3.53	0.171
	BD II	5 (38.5)	5 (29.4)		
	Unspecified BD	2 (15.4)	0 (0.0)		
Onset type (early onset)		10 (76.9)	17 (100.0)	4.36	0.037 *
Type of first episode	Depression	10 (76.9)	14 (87.5)	2.72	0.257
	Hypomania	2 (15.4)	0 (0.0)		
	Mania	1 (7.7)	2 (12.5)		
Employment status	Temporary sick leave	2 (16.7)	2 (12.5)	2.57	0.462
	Permanent sick leave	5 (41.7)	6 (37.5)		
	Retired	5 (41.7)	5 (31.3)		
	Active	0 (0.0)	3 (18.8)		
Family history of psychiatric disorders		6 (50.0)	13 (76.5)	2.18	0.140
Suicidal ideation		8 (61.5)	9 (60.0)	0.01	0.934
Suicide attempts		4 (30.8)	5 (33.3)	0.02	0.885
Mood stabilizers		12 (92.3)	15 (93.8)	0.02	0.879
Antipsychotics		9 (69.2)	10 (62.5)	0.14	0.705
Antidepressants		6 (46.2)	8 (50.0)	0.04	0.837
Benzodiazepines		4 (30.8)	5 (33.3)	0.02	0.885

BD, Bipolar Disorder; CRASH, Cognitive Reserve Assessment Scale in Health; FAST, Functioning Assessment Short Test; HDRS, Hamilton Depression Rating Scale; YMRS, Young Mania Rating Scale. * Indicated statistically significant values.

## Data Availability

The data presented in this study are available on request from the corresponding authors.
